# New Genetic and Linguistic Analyses Show Ancient Human Influence on Baobab Evolution and Distribution in Australia

**DOI:** 10.1371/journal.pone.0119758

**Published:** 2015-04-01

**Authors:** Haripriya Rangan, Karen L. Bell, David A. Baum, Rachael Fowler, Patrick McConvell, Thomas Saunders, Stef Spronck, Christian A. Kull, Daniel J. Murphy

**Affiliations:** 1 Centre for Geography and Environmental Science, Monash University, Melbourne VIC, 3800, Australia; 2 Royal Botanic Gardens Melbourne, South Yarra, VIC, 3141, Australia; 3 Department of Botany, University of Wisconsin-Madison, Madison, WI, 53706, United States of America; 4 School of Language Studies, The Australian National University, Canberra, ACT, 0200, Australia; 5 Independent Researcher—Linguist, PO Box 10, Derby, WA, 6728, Australia; 6 Linguistics, School of Culture, History, and Language, The Australian National University, Canberra, ACT, 0200, Australia; 7 Linguistics, Katholieke Universiteit Leuven, 3000, Leuven, Belgium; 8 Institut de Géographie et Durabilité, Universitie de Lausanne, Lausanne, CH, 1015, Switzerland; University of Western Ontario, CANADA

## Abstract

This study investigates the role of human agency in the gene flow and geographical distribution of the Australian baobab, *Adansonia gregorii*. The genus *Adansonia* is a charismatic tree endemic to Africa, Madagascar, and northwest Australia that has long been valued by humans for its multiple uses. The distribution of genetic variation in baobabs in Africa has been partially attributed to human-mediated dispersal over millennia, but this relationship has never been investigated for the Australian species. We combined genetic and linguistic data to analyse geographic patterns of gene flow and movement of word-forms for *A*. *gregorii* in the Aboriginal languages of northwest Australia. Comprehensive assessment of genetic diversity showed weak geographic structure and high gene flow. Of potential dispersal vectors, humans were identified as most likely to have enabled gene flow across biogeographic barriers in northwest Australia. Genetic-linguistic analysis demonstrated congruence of gene flow patterns and directional movement of Aboriginal loanwords for *A*. *gregorii*. These findings, along with previous archaeobotanical evidence from the Late Pleistocene and Holocene, suggest that ancient humans significantly influenced the geographic distribution of *Adansonia* in northwest Australia.

## Introduction

The role of humans in shaping crop genetic diversity has always been considered an integral factor in the evolution of agriculture in various regions of the world [1–3]. Interdisciplinary research combining genetics, linguistics, and archaeobotany has further enhanced understanding of the geographic patterns of animal and crop domestication and subsequent diffusion by humans [1–3]. Yet, there is very little comparable research on how anthropogenic agency has influenced the evolution and distribution of uncultivated plants that, nonetheless, have a long history of human use [4–6]. A striking example is that of *Adansonia*, the baobab tree, an iconic genus endemic to Africa, Madagascar, and the Kimberley region of northwest Australia [7,8]. These giant, long-lived trees hold significant cultural symbolism and multipurpose value as sources of food, medicine, water storage, shelter, and raw material for artisanal products in all these places [9–17]. Although there is no evidence of baobabs being cultivated historically, the distribution of the African baobab species, *Adansonia digitata* L., has been closely linked to human dispersal and settlement patterns [18,19]. This association is also recognised by the diversity and borrowing of terms for baobabs between language groups in Africa [4, 16]. In contrast, previous research on the evolution and geographic distribution of the Australian baobab, *Adansonia gregorii* F. Muell., has been based on the assumption of long-term natural processes [7,8] without any significant influence of human agency. This assumption may have stemmed from the long-held view of Aboriginal Australia as a ‘continent of hunter-gatherers’ [20–24] where anthropogenic agency was limited to ‘fire-stick farming’ of landscapes for nomadic foraging and hunting [25,26]. We explore the role of humans in shaping the evolution of *A*. *gregorii* by determining whether the geographic distribution of genetic diversity is explained, in part, by patterns of human migration, as inferred from linguistic analysis.

Levels of genetic divergence show that *A*. *gregorii* separated from other *Adansonia* species more recently than the break-up of Gondwana, but before the arrival of humans in Australia [7,8]. Leong-Pock Tsy et al. [27] demonstrated that *A*. *digitata* seeds retain their viability in seawater, making oceanic current dispersal feasible. From this it can be inferred that *A*. *gregorii* has been in Australia for longer than humans. There is also the possibility, albeit a less parsimonious explanation, that the species arrived more recently from an unknown population which is now extinct. One hypothesis outlining how *A*. *gregorii* may have arrived in Australia with humans has been explored in more detail by Pettigrew [28].

*Adansonia gregorii*, known in Australia as ‘boab’, is mainly distributed across the Kimberley region of northwest Australia, with a small extension eastward into the Victoria River District in the Northern Territory. The distribution of *A*. *gregorii* in the Kimberley extends from the northern coastline to the edge of the Great Sandy Desert and the Tanami Desert [17,29–32]. The Kimberley region represents the westernmost extent of the Australian Monsoon Tropics (AMT), which is characterised by highly seasonal rainfall and savanna vegetation [17,33]. The tree has been introduced more recently in urban centers of northern Australia for ornamental purposes [17].

The AMT biome is bounded to the south by arid habitats, which began developing in the Late Cenozoic and contain distinctly different biota [34,35]. The major biogeographical divide in northwest Australia is between the Kimberley to the west and Arnhem Land to the east, with more localized and specific barriers created by major river drainage systems [36–39]. Within the Kimberley, phylogeographic patterns for rock-wallabies (*Petrogale* spp.) and other species suggest an East-West Divide running through Central Kimberley [34,37,39]. Despite evidence of biogeographic barriers, a previously detailed population genetic analysis of *A*. *gregorii* has demonstrated that there is little genetic structure, with *F*_*ST*_ values non-significant between most populations [40]. Low geographic structure could be explained by a relatively recent arrival in the Kimberley, a recent genetic bottleneck, or high dispersal rates across the species’ range. For reasons detailed in Bell et al. [40], high dispersal is the most likely explanation.

In this paper we sought to evaluate the latter hypothesis – that the low levels of genetic structure within *A*. *gregorii* are due to high levels of gene flow and, specifically, that human-mediated seed dispersal has been an important evolutionary factor in the history of this species. Pollination in *A*. *gregorii* might occasionally entail birds or bats, but it appears that hawkmoths are the major pollinating agents [17,29,33,41,42]. Pollination-mediated gene flow is limited to the paternal genome and, in insect pollinated species, is often a less effective mechanism of long-distance gene flow than fruit dispersal [43–45]. Floodwaters could explain some fruit dispersal [7,16], but would probably not spread seeds beyond the edges of seasonal waterways and alluvial flats due to the fragile and dehiscent nature of the *A*. *gregorii* pericarp [7,33]. Other seed dispersing agents could be mammals such as rock wallabies (*Petrogale* spp.), other wallabies and kangaroos (*Macropus* spp.), which eat the fruit and disperse the seeds in their scat [17]. However, phylogeographic studies of the short-eared rock-wallaby (*P*. *brachyotis*), showed strong genetic structure, suggesting that at least this species has limited dispersal ability across biogeographic barriers in the Kimberley [39,46].

Anthropogenic agency has not been formally considered as a gene flow vector for *A*. *gregorii*, despite archaeological evidence of its long-term use by Aboriginal groups in the region [28,47–50]. This omission may be due to the fact that the boab was not cultivated historically, or not considered part of the food crops traditionally harvested by Aboriginal groups. Also, the presence of boab fruit pod remains at one or two archaeological sites is not sufficient to demonstrate anthropogenic dispersal across the species’ geographic range. Additional evidence is needed to evaluate whether humans have played a role in boab gene flow. We reasoned that historical linguistics, which has been used in combination with other sources of data to trace geographic patterns of diffusion of domesticated species [1–3], could be applied to a non-domesticated tree species such as *A*. *gregorii* [6]. Specifically, in this study, we investigate the role of human agency in the gene flow of *A*. *gregorii* by testing for congruence between the spatial distribution of genetic variation in *A*. *gregorii* trees and associated word-forms in the Aboriginal languages of northwest Australia. We demonstrate a high level of spatial overlap between the genetic and linguistic data. Our results indicate that, as previously shown for *A*. *digitata* in Africa [19,51,52], ancient humans have influenced the distribution of genetic diversity of *A*. *gregorii* in northern Australia, probably by acting as seed dispersal agents over long distances.

## Materials and Methods

This study makes use of two recently published data sets relating to *A*. *gregorii* from different fields of research. The first of these is genetic data from six nuclear microsatellite loci, of 220 *A*. *gregorii* individuals [40] (S1 Dataset). Although the number of microsatellite loci is low and can render some quantitative methods inaccurate, we used this data to make qualitative observations on patterns of dispersal and relationship between populations. The second data set consists of the words used for the boab tree in Aboriginal languages from across the species range [53] (S2 Dataset). This study brings these two datasets together, analysing them simultaneously to detect any congruent patterns.

### Phylogenetic analysis of microsatellite data

**Fig 1:**
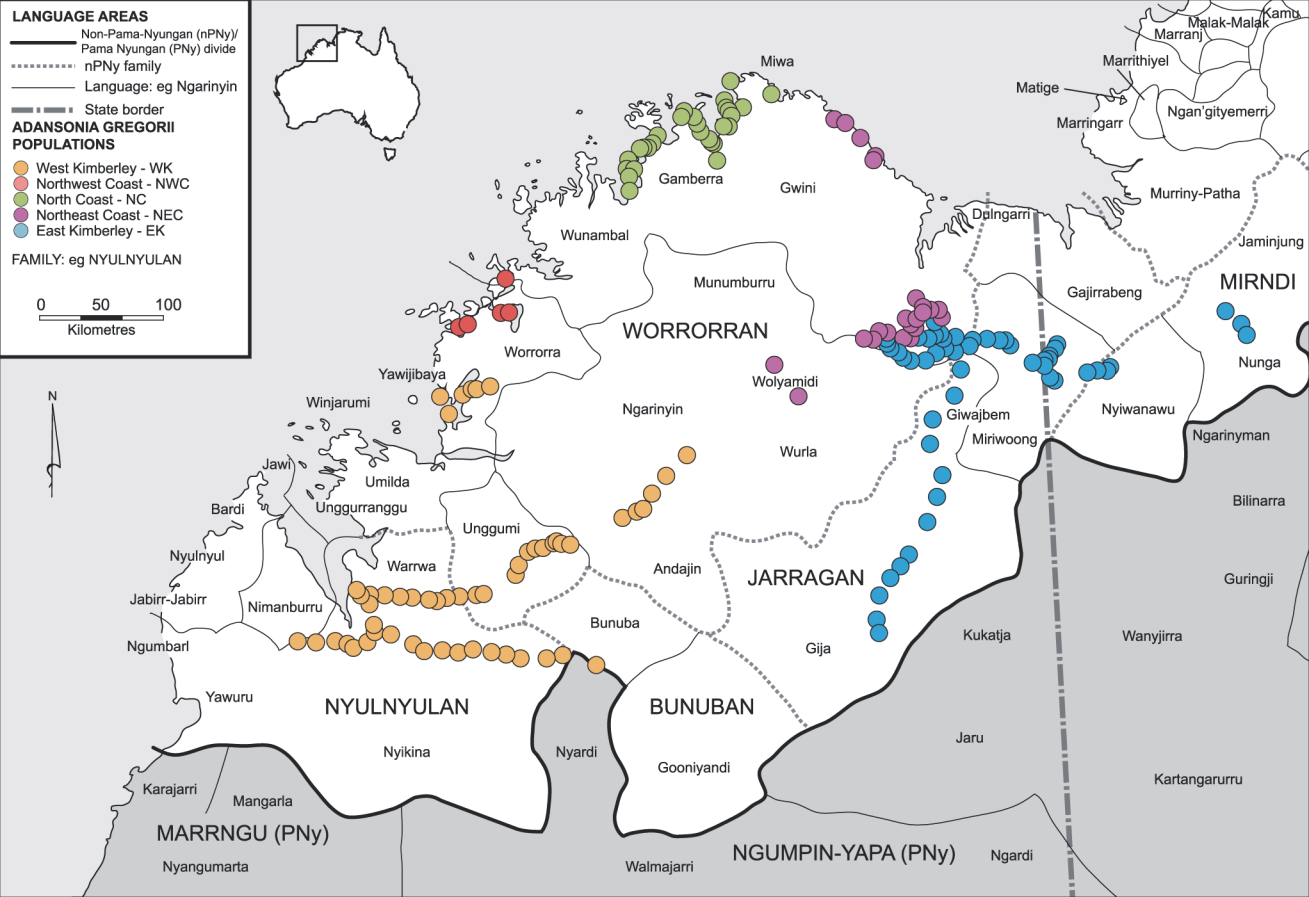
Graphical representation of the five inferred *Adansonia gregorii* populations from the sPCA, shown on a map of Aboriginal language areas of the Kimberley region. Map based on Harvey [58] and McGregor and Rumsey [63].

We conducted phylogenetic analysis of the *A*. *gregorii* populations shown in Fig. 1, using previously obtained microsatellite data [40]. Collection of plant material, with appropriate permissions, deposition of specimens, and laboratory analyses are described in Bell et al. [40]. This study used the spatial principal components analysis (sPCA) to map and define genetic populations, which identified five genetically differentiated clusters: West Kimberley (WK); East Kimberley (EK); North West Coast (NWC), North Coast (NC), and North East Coast (NEC). Genetic divergence between these clusters was weak, but statistically significant [40], hence these were treated as five populations for subsequent analyses.

To obtain an estimate of the relationships among the five populations, we first obtained 1000 bootstrap replicates of the microsatellite data. We calculated pairwise genetic distances between populations with the *D*_*C*_ method [54] for each bootstrap replicate in Phylip v 3.69 [55]. These distance matrices were then used to construct unrooted neighbour-joining trees, and a bootstrap consensus tree in Phylip. While more complex methods have been found to provide greater accuracy in dating divergence events from microsatellite data, the simple *D*_*C*_ method was judged suitable because it performs as well as these methods at phylogenetic reconstruction [56]. The resulting tree was midpoint rooted.

### Phylogeny of linguistic data

The Aboriginal languages of Australia are broadly classified into a number of families, Pama-Nyungan (***PNy***) and ten or more non-Pama-Nyungan (***nPNy***) families [57,58]. The ***PNy*** language groups are most widespread across the continent, while the ***nPNy*** families (excluding Tasmania) exist primarily in northwest Australia across the Kimberley and western Arnhem Land [58,59]. The ***PNy*** language subgroups and ***nPNy*** families have been distinguished by the comparative method [60], including grammatical morphology and measures of difference in vocabulary [57], with sub-groups identified by sets of ‘shared innovations’ [61]. A Neighbour Joining analysis of twenty-one Kimberley languages using the method described in Hudson and Bryant [62] was published by McGregor and Rumsey [63]. The tree generated from that analysis showed the relationships between these Kimberley languages based on lexical resemblances from a basic wordlist of 105 meanings, containing minimum numbers of loanwords [63]. A greater degree of proximity and shared branches in the phylogeny indicates higher lexical similarity between the respective languages. We used this neighbour-joining tree in conjunction with a more general consensus about family classification using the comparative method in discussion of the origins of words for the boab tree [64].

### Determining boab proto-words, inherited forms, and loanwords

Data on boab word-forms and related terminology was drawn from both published and unpublished sources and transliterated into a standard orthography [53] (S2 Dataset). The distinction between inheritance (vertical transmission) and borrowing (horizontal transmission) of word-forms is vital to our analysis. Proto-words are word-forms that can be traced back through successive, plausible steps to a common ancestral word-form in an ancestral language (known as the proto-language) [60]. In vertical transmission, proto-words are inherited through nodes in a phylogeny to a number of more recent descendant languages, often changing meaning and form over time. Horizontal transmission, or diffusion, occurs through borrowing, whereby words enter and become adopted in other languages that may or may not be closely related by inheritance. There are several types of evidence used to detect loanwords and their original source [6]. Borrowed words in a language are ones that cannot be connected to plausible proto-words in that language but instead show strong similarity to words in another language. The identification of a loanword is further corroborated when the word can be analyzed into meaningful word-parts in the other language (the putative loan source) but not in a language into which it has been borrowed.

### Genetic-Linguistic Analysis

The spatial distribution of each language was defined following Harvey [58] and McGregor and Rumsey [63]. The *A*. *gregorii* distribution includes five ***nPNy*** families (Worrorran, Nyulnyulan, Bunuban, Jarragan, and Mirndi) and two ***PNy*** language subgroups (Marrngu and Ngumpin-Yapa) (Fig. 1). Three sets of relationships of genetic and linguistic data for *A*. *gregorii* were examined for evidence of human-mediated dispersal across the plant’s geographic range.

#### Boab populations and language families

**Fig 2:**
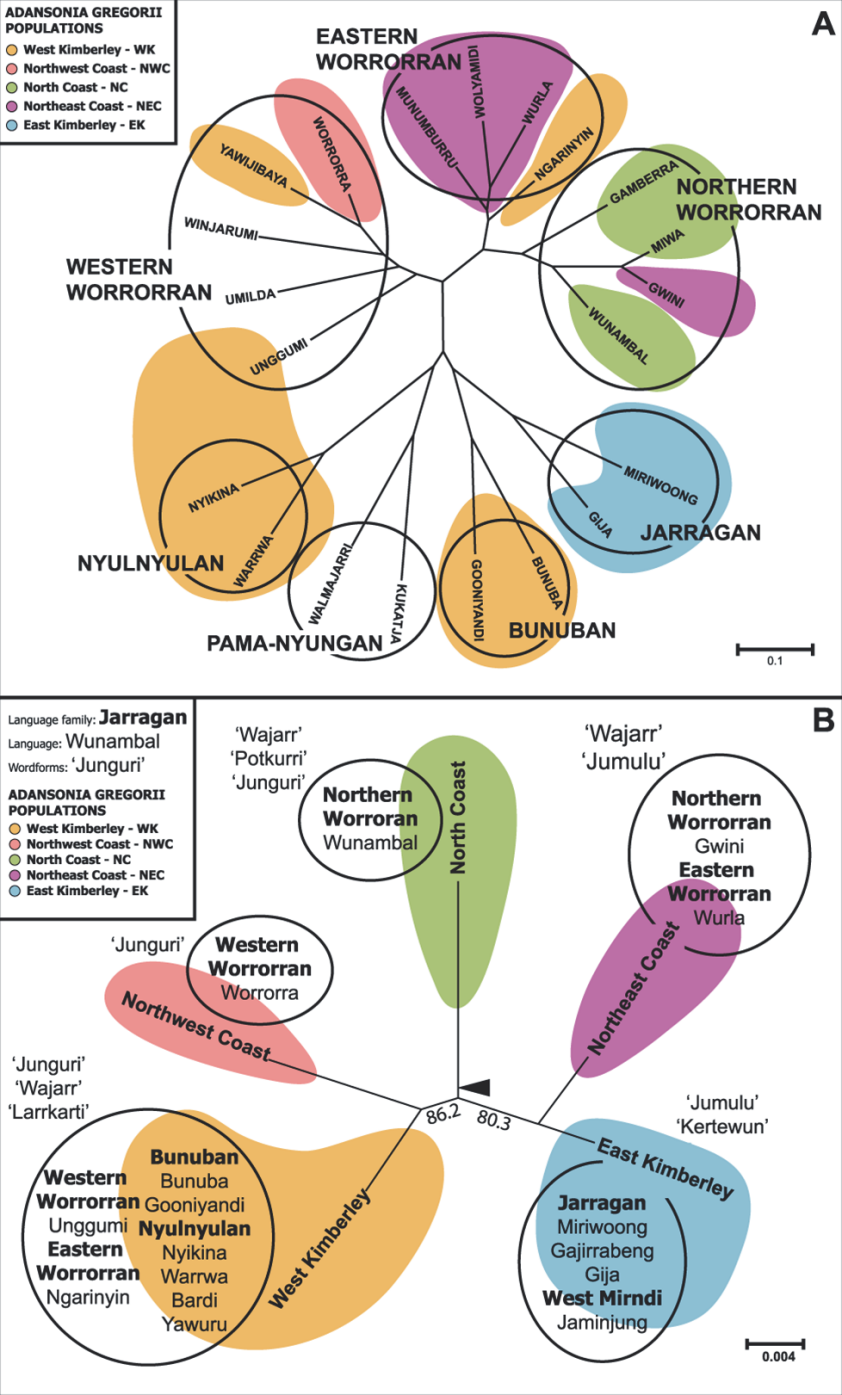
A. Neighbour-joining tree of lexical resemblance among Worrorran and nearby Aboriginal languages of the Kimberley, following McGregor and Rumsey [63]. Twenty-one languages (19 belonging to ***nPNy*** families and 2 belonging to the Ngumpin ***PNy*** sub-group) were chosen for analysis on the basis of availability of relatively reliable information. Distance between nodes indicates degree of dissimilarity between the languages according to the scale shown. The five inferred *A*. *gregorii* populations have been superimposed on the tree to visualize spatial congruence with language groups. **B. Neighbour-joining phylogeny of the five inferred *A*. *gregorii* populations showing their occurrence in language group areas according to Harvey [58].** Distance between nodes indicates genetic divergence between populations calculated with the *D*_*C*_ method. Arrow indicates mid-point. The word-forms for boabs associated with each of the five inferred *A*. *gregorii* populations are shown in quotation marks.

Geographic congruence between *A*. *gregorii* populations and language areas was examined by superimposing the genetic data on the phylogenetic tree of the main Kimberley language groups analysed by McGregor and Rumsey [63], and conversely, by superimposing the language group areas [58] in which the boab populations occur on the neighbour-joining tree of the five genetic populations (Fig. 2A and B). A high level of geographic congruence between the *A*. *gregorii* genetic populations and the language areas they occupy would be consistent with the idea that people have moved boabs extensively within language areas, but not so much between language areas.

Analysis of molecular variance (AMOVA [65]) was used to test whether there was a significant broad scale correlation between genetics and language family area. Boab individuals were grouped according to their occurrence in the nPNy family areas and significance was tested with 999 permutations.

A partial Mantel test of genetic distance vs. language family area with geographic distance as a covariate [66] was used to test whether any statistical significance inferred by the AMOVA was a result of isolation by distance in both language and genetic variation. Genetic distance was calculated between boab individuals using the *D*_*C*_ method [54], and boab individuals were assigned to language family areas as described above.

We used a permutation test, implemented in Mesquite [67], to test for statistically significant association between language groups and genetic identity. The genetic clusters were scored as single, multistate characters. The length of this character on the language phylogenetic tree was calculated under equally-weighted parsimony. We permuted this character 999 times, and calculated its length on the language phylogenetic tree. We then determined whether the length of the original character was inside the expected distribution based on the randomly permuted character.

#### Language families and boab word-forms

To examine the geographic relationship between boab word-forms and language families, the numbers of boab word-forms in each of the ***nPNy*** language families were examined to identify languages with higher and lower numbers of boab word-forms (Table 1). A greater diversity of word-forms for *A*. *gregorii* within language family areas may suggest long-term presence of, and interaction with, boab populations. Conversely, lower diversity of word-forms for the tree could be either due to recent rapid expansion of a language family area, or to a more recent interaction of those populations with boabs.

**Table 1:** Word-forms for *Adansonia gregorii* in the Kimberley region.

**Section A: Word-form for boab tree**					
**Non-Pama- Nyungan Family, Pama-Nyungan (*PNy*) subgroup**	**Language group**	**Word-Form for boab tree**	**Reconstructed/loan-source form**	**Original meaning**	
N. Worrorran	Wunambal	junguri, jungeri	junguri		
N. Worrorran	Wunambal	po:rkuru	potkurri		
N. Worrorran	Wunambal	potkurri	potkurri		
N. Worrorran	Wunambal	wajer	wajarr		
N. Worrorran	Gwini	jumulu	jumulu		
W. Worrorran	Worrorra	jungura	junguri		
W. Worrorran	Worrorra	jungurim	junguri		
W. Worrorran	Unggumi	la:kaji	larrkarti		
E. Worrorran	Wurla	wajarr	wajarr		
E. Worrorran	Ngarinyin	junguri	junguri		
E. Worrorran	Ngarinyin	jungulan	junguri?		
E. Worrorran	Ngarinyin	larrkarri (larrkari)	larrkarti		
Bunuban	Gooniyandi	wajarri	wajarr		
Bunuban	Bunuba	larrkari	larrkarti		
Nyulnyulan	Nyikina	larrkati	larrkarti		
Nyulnyulan	Warrwa	larrkarti	larrkarti		
Nyulnyulan	Bardi	larrkiti	larrkarti		
Nyulnyulan	Yawuru	larrkarti	larrkarti		
Marrngu (*PNy*)	Karajarri	larrkarti	larrkarti		
Marrngu (*PNy*)	Mangarla	larrka-rti	larrkarti	‘split-prone to’	
Marrngu (*PNy*)	Nyangumarta	larrkarti	larrkarti		
Ngumpin (*PNy*)	Walmajarri	larrkarti	larrkarti		
Ngumpin (*PNy*)	Jaru	larrkarti	larrkarti		
Ngumpin (*PNy*)	Jaru	jamarlu	jumulu		
Jarragan	Gajirrabeng	kertewun	kertewun		
Jarragan	Miriwoong	katawun	kertewun	? ‘egg’	
Jarragan	Gija	jumulu-ny	jumulu		
Jarragan	Gija	kuwulu-ny, tyaru-ku-ny	kulpe: ‘tree’, jare: ‘stomach’	‘big bellied tree’	
W. Mirndi	Jaminjung	kuruwuny (kuruwan)	kertewun		
Ngumpin (*PNy*)	Bilinarra	jamula-ng	Jumulu		
Ngumpin (*PNy*)	Ngarinyman	jamula-ng (jamurlang)	jumulu		
**Section B: Word-forms for boab tree parts**					
**Non-Pama- Nyungan Family, Pama- Nyungan (*PNy*) subgroup**	**Language group**	**Word-Forms for boab tree parts**	**Reconstructed/ loan source form**	**Translation**	**Original meaning**
W. Worrorran	Worrorra	yu:ku	yuukun	dry boab fruit pod	
W. Worrorran	Unggumi	lakeri	larrkarti	boab fruit pod	
E. Worrorran	Ngarinyin	yu:kun	yuukun	dry boab fruit pod	
E. Worrorran	Ngarinyin	irrke	?	pith of the boab tree	
E. Worrorran	Ngarinyin	kuranpun	?	pith of the boab tree	
Bunuban	Bunuba	wajarr	wajarr	boab fruit pod	
Bunuban	Bunuba	ngipi	ngipi (loanword from Nyikina)	boab seeds	
Nyulnyulan	Nyikina	nguja		dry boab fruit pod	
Nyulnyulan	Nyikina	ngipi		boab seeds	
Jarragan	Miriwoong	jang-nge-ng	jang ‘eat’	edible pith of fruit pod	‘eating-for’
Jarragan	Gajirrabeng	tijperu-ng	?	seed inside boab fruit pod	Possibly related to tij- ‘dead’
Jarragan	Gajirrabeng	murl-ng	murl	boab seed	‘eye’
Jarragan	Gija	larrkarti-m	larrkarti	boab fruit pod	
Jarragan	Gija	jililiny	?	boab seed	
Jarragan	Gija	wawang-ku-ny	?	inside of boab fruit pod	?
Jarragan	Gija	jang-nge	jang-nge	edible pith of fruit pod	‘eating-for’
W. Mirndi	Jaminjung	jangi	jang-nge	edible pith of fruit pod	
Ngumpin (PNy)	Ngarinyman	jangi	jang-nge	edible pith of fruit pod	

Data on boab word-forms and related terminology were drawn from both published and unpublished sources (S2 Dataset).The two left-hand columns identify the non-Pama-Nyungan language family and Pama-Nyungan (*PNy*) sub-groups, and the associated language groups. The word-forms are represented in a standardized orthography. A hyphen indicates a morpheme break, e.g., separating a stem from a suffix. The subsequent columns list the reconstructed or loan-source form and the probable original meaning of the reconstructed source form. Section A lists word-forms for the boab tree; Section B lists word-forms for boab tree-parts.

#### Boab word-forms and genetic population phylogeny

If humans have played a major role in dispersing boabs over the Kimberley, then we might expect congruence between diffusion of loanwords and dispersal of genotypes. Gene flow between *A*. *gregorii* populations has previously been examined based on coalescent analysis, using IMa2 [ 40 ]. That analysis yielded estimates of the relative migration rates in two major directions: between western and eastern populations and between coastal and inland populations [40]. These directional patterns of gene flow were compared with the directional movement of boab word-forms, especially loanwords, using the comparative method [6, 64] to trace their original source and examine patterns of geographic congruence. The reconstructed loan source forms for the boab tree in Table 1 were used to identify five words which showed directional movement of borrowing between the languages of the Kimberley region.

## Results

### Phylogeny of *A*. *gregorii* populations

Phylogenetic analysis generated an unrooted tree for the five inferred populations with bootstrap support values greater than 80% for both internal branches (Fig. 2B). Midpoint rooting suggests that the WK and NWC populations are sisters, as are the EK and NEC populations, with the deepest divergence separating the NC population from the other four. However, since the root is close to the node, this conclusion should be treated with caution.

### Boab populations and language family areas

The association of the five *A*. *gregorii* populations on the language phylogenetic tree (Fig. 2A) and vice-versa, of language families with boab population phylogeny (Fig. 2B), did not reveal strict geographic congruence. Statistical analyses showed that genetic variance between boab individuals from different ***nPNy*** family areas was low but statistically significant (AMOVA, 3% of total genetic variation, *P* = 0.001). A partial Mantel test of genetic distance vs. language family area with geographic distance as a covariate was not significant (*R*_*xy*_ = 0.00728; *P* = 0.285), indicating that the statistical significance of the AMOVA could be due to spatial autocorrelation in both languages and genetic variation. However, it is noteworthy that there seems to be a geographically sharp distinction between the EK and NEC genetic clusters, and this break coincides with the distinction between the Jarragan and Worrorran language families.

The mapping of genetic population assignments onto the language tree required 4 steps for the original, unpermuted data whereas the permuted characters required from 5 to 10 steps, with a mean of 8.55. With 999 permutations, this implies a p-value ≤ 0.001, indicating a strongly non-random pattern of association between the language tree and the genetic clusters. Because geography was not included as a covariate, we cannot rule out the possibility that this association is driven by geography rather than a causal relationship between boab genetics and human language variation.

### Language family areas and *A*. *gregorii* word-forms

The greatest diversity of word-forms for *A*. *gregorii* is found in the northern coastal areas encompassing the Worrorran language family, followed by those in the Jarragan family (Table 1 and Fig. 2A). Each has one dominant word for the tree species that is not borrowed from elsewhere. Worrorran has the term *junguri*, and Jarragan has *jumulu*. Both terms are reconstructable as proto-words for boab in these language families. In addition to these proto-words and their descendant forms, the language families have other inherited word-forms for boabs. Worrorran (Northern) has the forms *potkurri* and *wajarr* in the Wunambal language; the former word is restricted to this specific language, while the latter is also used in Wurla (Eastern Worrorran). *Wajarr* has either been borrowed into the Bunuban family or from it: evidence is equivocal at this stage. Forms of the Jarragan proto-word *jumulu* have been borrowed into northern Worrorran and into the ***PNy*** Ngumpin subgroup. Jarragan also has the term *kertewun* that has been borrowed further east into the ***nPNy*** Mirndi language family.

Of particular interest is the boab word-form *larrkarti*, which is from a language subgroup associated with the desert area south of the main bioregions of *A*. *gregorii* distribution. Analyzability of this word into two morphemes (*larr*-split; *karti*-side) suggests that it comes from the Karajarri (and/or the closely related and neighbouring Mangarla) languages of the ***PNy*** Marrngu subgroup on the southwest periphery of the Kimberley and is of relatively recent origin [53]. While both the elements *larr* ‘split lengthways’ and the suffix –*karti* ‘towards; side’ do occur in other languages in the region separately and with somewhat different meanings, their coincidence with Karajarri and Mangarla languages is a strong indication that this is the origin of the new coined term for the boab. As a whole, *larrkarti* means ‘split side’, which could be an allusion to the hollow trunks of old trees or the dehiscent nature of the boab fruit pod, or more likely to the manner in which the shelled fruit splits into longitudinal segments. The coining of this word may well have come about when the Marrngu speaking people moved from the desert into the west Kimberley areas where they would have encountered these unfamiliar trees in the landscape. This Marrngu word for boab has been borrowed and incorporated into the vocabularies of some of the ***nPNy*** Worrorran (Western and Eastern) – Bunuban – Nyulnyulan language families and one Jarragan language alongside other inherited word-forms. *Larrkarti* has also been borrowed into the ***PNy*** Ngumpin language subgroups from desert areas southeast of the Kimberley.

### Boab word-forms and genetic population phylogeny

Fig. 2B illustrates the overlap between *A*. *gregorii* words-forms and population phylogeny. The NC population, which appears to be sister to the remaining populations, has the inherited Worrorran boab word-forms *junguri* and *potkurri*, and possibly *wajarr*. The NWC population has the inherited form *junguri*. The WK population, which clusters with NWC and is distributed across a number of ***nPNy*** language family areas has three words, *junguri*, *wajarr*, and *larrkarti*. The NEC population has *wajarr*, *jungulan* (a probable cognate of *junguri*) and the Jarragan loanword *jumulu*. The EK population, which occurs across the Jarragan and Mirndi families, has the word-forms *jumulu* and *kertewun*. Each pair of genetic clusters shares at least one boab word-form suggesting extensive word exchange.

### Gene flow and diffusion of boab loanwords

Statistically significant gene flow was recorded across all boab populations in the Kimberley [40]. Migration rates were estimated by grouping the populations across two axes: West (WK, NWC) ↔ East (NC, NEC, EK); and Coast (NWC, NC, NEC) ↔ Inland (WK, EK). The West→East migration rate was 0.47 individuals/ year and the East→West rate was 0.11. The Coast→Inland migration rate was 0.74, and Inland→Coast was 0.50 [40].

**Fig 3:**
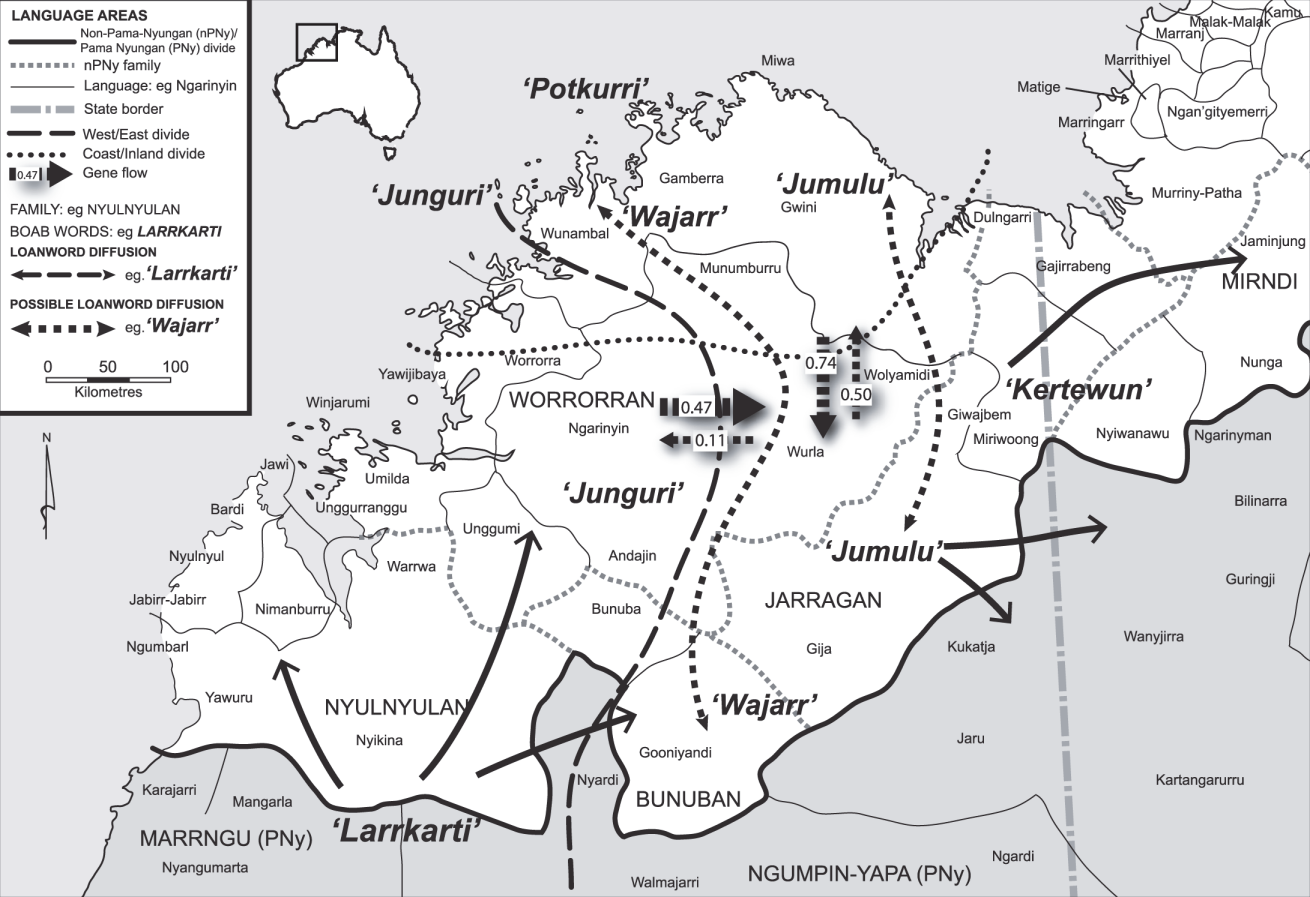
Gene flow between *A*. *gregorii* populations and boab loanword diffusion across the Kimberley region. Approximate locations of inferred East-West and Coast-Inland biogeographic divides are represented by dotted lines. Gene flow between East-West and Coast-Inland is calculated using IMa2 is in units of individuals/year, and shown with dashed block arrows. Unbroken arrows show the direction of borrowing of boab loanwords among the Kimberley languages; broken arrows show possible directions of loanword movements.

We compared these gene flow patterns with inferred loanword diffusion patterns (Fig. 3). The linguistic analysis of boab word diffusion is consistent with the existence of an East-West biogeographic divide [36–39, 40]: *jumulu* and *kertewun* have diffused further to the east, but not into west Kimberley, whereas the word *junguri* remains in the northwest and has not diffused into eastern Kimberley. *Larrkarti* has diffused widely from its inferred source in the southwest into western and central Kimberley languages, but not further into northern Worrorran languages.

If human-mediated dispersal were responsible for the East ↔West and Coast ↔ Inland dispersal, we might expect to see corresponding loanword diffusion along these axes. Furthermore, since there is evidence of much more west-to-east gene flow than east-to-west gene flow across the inferred East-West divide, we might expect more west-to-east diffusion of loanwords than the reverse. These directional patterns accord well with the diffusion of boab loanwords shown in Fig. 3. *Larrkarti* and *kertewun* have diffused West→East, but there are no cases of East→West boab loanword diffusion. Thus, the direction and rates of migration of *A*. *gregorii* gene flow show correspondence with the inferred diffusion of boab loanwords.

## Discussion

Our study supports the hypothesis that human-mediated dispersal has played a role in shaping the geographical distribution of *A*. *gregorii*. The limited morphological and genetic divergence of the *Adansonia* genus in northwest Australia, despite the presence of biogeographic barriers, can be attributed to high gene flow within *A*. *gregorii* [40]. Given the lack of other obvious seed dispersal agents, the lack of evidence of barriers to gene flow is most easily explained by a long history of humans moving boab seeds. Concordance between gene flow and loanword diffusion further supports the hypothesis of human-mediated dispersal of *A*. *gregorii*. Loanword movements across all boab populations provide an indication of patterns of interaction between ***nPNy*** language families and ***PNy*** subgroups that would have influenced the geographic distribution of *A*. *gregorii* in the Kimberley region.

Additional evidence of anthropogenic agency in facilitating boab gene flow comes from archaeological excavations at Carpenter’s Gap Shelter located in the Napier ranges of the Kimberley, which falls within the Bunuba language area [48,49]. Lithic and macrobotanical remains from this rockshelter site showed continuity of human occupation extending over 40 ka into the late Pleistocene. Carbon dates recorded boab pod fragments at 39 ka, 20 ka, 18 ka and 15 ka [48,49], with substantial increase in pod deposition from 3 ka onwards and peaking at around 650 years ago [49]. Old boab trees have been found near aboriginal middens in western Kimberley, providing further evidence of long-term human consumption of the fruit [48]. Prehistoric rock art in the northern Kimberley also shows possible depictions of the tree, indicating its cultural significance to ancient human groups that may have occupied this region [28].

Based on the above evidence, we postulate that recent boab evolution and geographic distribution have been shaped primarily through ancient human agency. The phylogenetic tree of boab populations and predominant direction of gene flow together imply that the source populations for *A*. *gregorii* dispersal were most likely in the extreme northwest Kimberley, potentially overlapping with the inferred NC population area. The range of this source population is likely to have extended beyond the current coastline at the Last Glacial Maximum (LGM, roughly 20 ka) when sea levels were over 120 m below present-day, and the northwest continental shelf was exposed to the maximum extent [68]. The increased land surface exposure of both Sahul and Sundaland shelfs, lower sea surface temperatures (SST), and altered oceanic currents due to closure of several shallow seas and passageways between these continental shelves contributed to a northward shift of the Inter-Tropical Convergence Zone (ITCZ), thereby reducing seasonal precipitation levels [69] and creating semi-arid savanna conditions [70] in which ancestral boab populations would have existed in northwest Australia.

**Fig 4:**
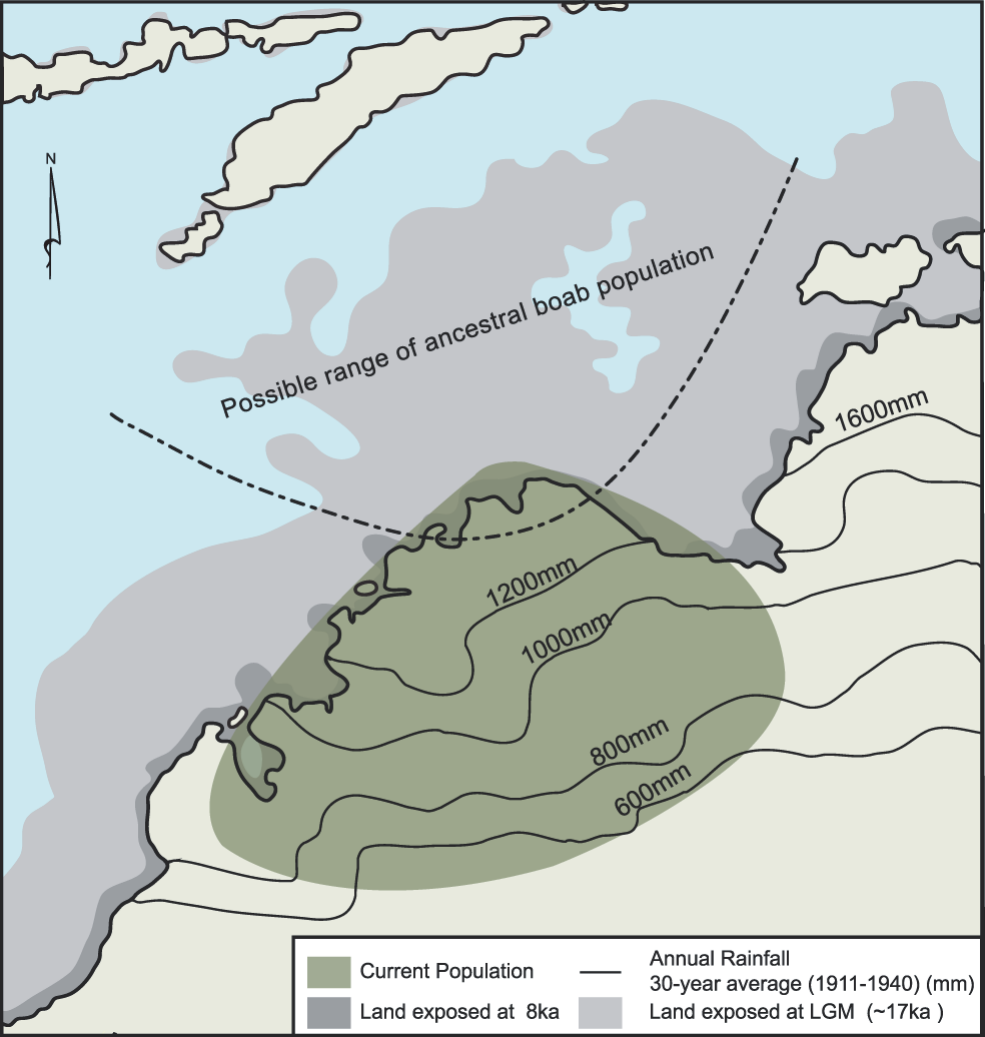
Sketch of LGM scenario showing land exposed at 17 ka and at 8 ka in relation to present-day coastlines, based on Coller [87]. The possible extent of ancestral boab population distribution on the exposed continental shelf is shown by a dashed arc; current boab distribution shown in light grey. Rainfall isohyets (mm) are based on 30-year annual average rainfall (1911–1940) estimates obtained from the Australian Bureau of Meteorology [88].

Fig. 4 sketches a possible LGM scenario for this boab population distribution, showing land exposed at 17 ka and at 8 ka in relation to present-day coastlines. With rainfall as much as 30 to 50% below present day levels [69] and higher levels of aeolian landform activity [71], subtropical desert conditions would have prevailed across much of the exposed continental shelf beyond present-day Kimberley [72–75]. These arid climatic conditions, particularly the low levels of seasonal rainfall, would have limited the distribution of *A*. *gregorii*, as the current distribution of the species coincides with areas receiving at least 700 mm of annual seasonal rainfall [76]. Therefore, under drier climatic conditions during the LGM due to the northward shift of the ITCZ [69], it is likely that boab populations would have been limited to the extreme northern coast of present-day Kimberley and the exposed continental shelf.

Subsequent sea-level rise and restoration of monsoonal activity during the post-glacial period and the Pleistocene-Holocene transition between 17 and 6 ka flooded the Sahul shelf and established the present-day coastlines of northern Australia [68,73]. The flooding of the continental shelf beyond the present-day Kimberley coast, along with increased monsoonal rainfall over inland Kimberley, would have altered the distribution of ancestral *A*. *gregorii* populations and possibly created a genetic bottleneck from which current populations would have expanded [40]. We propose that ancient human groups that lived on the coast along this previously exposed shelf during the Late Pleistocene would have retreated from inundated areas and carried boab fruit with them as they migrated further south and east.

Late Pleistocene records of boab remnants at the Carpenter’s Gap archaeological site [48,49] may represent sporadic visits of human settlers from the north [77]. The site record of the presence of shells and beads from the Early Holocene suggests movement of high value goods from the coast [78]. In common with other Australian archaeological sites [79], evidence for occupation at Carpenter’s Gap increases sharply from mid- to late Holocene, perhaps reflecting a demographic expansion in southern Kimberley [79]. This population increase, combined with other factors such as climatic and vegetation change [49,50], growth in local boab populations, and more frequent use of the site for cultural ceremonies and exchanges [77] could explain the increase in boab pod remnants along with other food plants and seeds from about 3000 to 650 years ago [48].

The increased mobility of Aboriginal groups in the southern Kimberley during the Late Holocene is likely to have been influenced by greater climate variability in northern Australia. In contrast to the Early Holocene, the Late Holocene (∼1000 BCE – 500 CE) climate in the Australian Monsoon Tropics was marked by periods of increased seasonality and aridity [80–82]. These conditions may have contributed to increased mobility of Aboriginal groups between different parts of the Kimberley and would have contributed to higher *A*. *gregorii* gene flow through fruit and seed dispersal, accompanied by diffusion of associated word-forms.

The linguistic data provides additional indication of patterns of migration and social interaction that could have contributed to the spread of *A*. *gregorii* (Table 1 and Fig. 3). The periods of aridity during the Late Holocene may have affected the survival of desert-based Marrngu (***PNy*** subgroup) speakers and led to their migration into southern and western Kimberley. The word-form *larrkarti* was probably coined by Marrngu speakers during this period when they would have encountered the boab tree in the Kimberley landscape. At the same time, the mobility and cultural interactions of these ***PNy*** groups with neighbouring ***nPNy*** language groups such as Nyikina and Bunuba would have increased. Evidence that the diffusion of the loanword *larrkarti* is of a relatively recent nature is demonstrated in the way it has been incorporated into other languages, generally in an unchanged form [61]. The Gija language (southern Jarragan) has retained its inherited word *jumulu* for the tree and adopted *larrkarti* for the fruit pod, perhaps indicating the salience and portability of the edible seed pod in the more recent borrowing. Likewise, the Jaru language (***PNy*** Ngumpin subgroup) has no inherited words for boab and uses both *jamula* (modified from Gija) and *larrkarti* for the tree. Other examples of recent boab expansion and loanword diffusion further east can be found in the Ngarinyman language (Ngumpin) in the Northern Territory, where the words *jang-nge* (borrowed from Miriwoong, meaning ‘for eating’) and *jumulu* (borrowed from Gija) are used for the fruit or its edible pith, and the tree respectively.

The possible climatic influences on human migration and boab loanword movement in the Kimberley echoes some aspects of Bostoen et al.’s [6] description of climate-induced dynamics and Bantu expansion in Africa. Although there is no analogous evidence of a large-scale expansion of a single linguistic group into the Kimberley, the ***PNy*** Marrngu term *larrkarti* and other pre-existing ***nPNy*** language words such as *wajarr*, *jumulu*, and *kertewun* moved across the Kimberley in patterns corresponding with multi-directional boab gene flow as shown in our study.

The high gene flow in *A*. *gregorii* appears similar to the case of *A*. *digitata* in Africa, where human agency has been involved in dispersal of the species [19,51,52]. The boab loanword movements in the Kimberley may be compared with Blench’s [4] account of the spread of Bantu words for *A*. *digitata*. He notes that the genetic diversity of baobabs in the ecological zones of West Africa and the diversity of vernacular names for the tree in African languages suggests considerable antiquity as well as significant east-west movement along trade routes and exchanges of associated ideas and terminology. However, despite the diversity of baobab names, he points out that two competing Bantu roots, #*mbuyu* and #*muramba*, and variations of these, are found in the Bantu languages of southern and eastern Africa. The Bantu expansion from the tropical forest areas of West Africa is said to have begun from around the middle of the first millennium BC (∼ 2500 BP onwards) and reached southern Africa by about 500 CE [6,83]. Blench argues that the Bantu would not have been familiar with the baobab because it does not grow in the tropical forest areas of Cameroon, Gabon and Congo where this proto-language group are thought to have originated. They would have encountered the tree as they expanded eastwards and emerged into the savanna, and developed new terms by either borrowing from resident hunter-gatherer groups or comparing it with some other tree species they already knew. The loan or variations of baobab words *mbuyu* and *muramba* in the Bantu languages of eastern and southern Africa would thus indicate the movement of Bantu into these areas [4]. The lower levels of genetic diversity of *A*. *digitata* in eastern and southern Africa detected by Leong Pock Tsy et al. [27] may likely be due to the Bantu expansion over this 3000 year period and their contribution to high levels of gene flow in baobabs across these regions.

## Conclusion

Our study demonstrates that the limited intraspecific divergence within *A*. *gregorii* in Australia is most likely due to high gene flow mediated by human agency, similar to that inferred for *A*. *digitata* in continental Africa [27, 51], combined with shifts in suitable habitat and a weak bottleneck following the end of the LGM [40]. Human use of *Adansonia* over many thousands of years in both continents would have contributed to gene flow over long distances and across biogeographical barriers. In contrast, it could be that the divergence of *Adansonia* into six species in Madagascar was possible in part because of the lack of humans until ca. 2 ka. However, this hypothesis can only be tested by further investigation of the ecological, physiological and biogeographic processes contributing to speciation within the *Adansonia* clade from Madagascar.

This study contributes new evidence for the role of ancient humans in influencing the evolution and distribution of non-domesticated plant species in Australia. Australia has long been viewed as a continent of hunter-gatherers [84] where prehistoric and Aboriginal populations prior to European colonisation played minimal roles in selecting and dispersing useful plants [23,24]. However, some recent studies have challenged this assumption by providing evidence for the role of ancient Aboriginal groups in the dispersal of food plants across the continent [77,85,86]. These include bananas (*Musa* spp.), taro (*Colocasia esculenta*) and some yams (*Dioscorea* spp.) in northern Australia [23], *Livistona* palms in Central Australia [85], and yam daisy (*Microseris scapigera*) in southeastern Australia [86]. Our findings add new insights regarding the role of ancient human agency in influencing the evolution and distribution of the boab, an important non-cultivated food plant species that has shaped the long-term landscape and environmental history of northwest Australia.

## Supporting Information

S1 Dataset Genetic data from six nuclear microsatellite loci of 220 *A*. *gregorii* individuals. (XLS)

S2 Dataset Terms for boab in Aboriginal languages of the Kimberley region. (XLS)
