# Correction: New Genetic and Linguistic Analyses Show Ancient Human Influence on Baobab Evolution and Distribution in Australia

**DOI:** 10.1371/journal.pone.0127582

**Published:** 2015-04-27

**Authors:** Haripriya Rangan, Karen L. Bell, David A. Baum, Rachael Fowler, Patrick McConvell, Thomas Saunders, Stef Spronck, Christian A. Kull, Daniel J. Murphy

The following information is missing from the Funding section: DB acknowledges NSF grant 1354793. The complete, correct funding information is as follows: The genetics and biogeographic research in this paper was funded by an Australian Research Council grant (DP1093100, awarded to Rangan, Kull, and Murphy); the linguistic research was supported by a grant from the Kimberley Foundation Australia (awarded to McConvell), a US NSF Grant (HSD 902114, awarded to Bowern, et al., including McConvell), and an AIATSIS grant (G2011/76, awarded to Spronck). DB acknowledges NSF grant 1354793.
